# Modeling of Eddy Current Welding of Rail: Three-Dimensional Simulation

**DOI:** 10.3390/e22090947

**Published:** 2020-08-28

**Authors:** Xiankun Sun, He Liu, Wanqing Song, Francesco Villecco

**Affiliations:** 1 School of Electronic and Electrical Engineering, Shanghai University of Engineering Science, Shanghai 201620, China; M020318178@sues.edu.cn (H.L.); swqing@sues.edu.cn (W.S.); 2 Department of Industrial Engineering, University of Salerno Via Giovanni Paolo II 132, 84084 Fisciano, Italy; fvillecco@unisa.it

**Keywords:** Three-dimensional model, rail welding, eddy heating, grid planning, electromagnetic induction

## Abstract

In this paper is given a three-dimensional numerical simulation of the eddy current welding of rails where the longitudinal two directions are not ignored. In fact, usually it is considered a model where, in the two-dimensional numerical simulation of rail heat treatment, the longitudinal directions are ignored for the magnetic induction strength and temperature, and only the axial calculation is performed. Therefore, we propose the electromagnetic-thermal coupled three-dimensional model of eddy current welding. The induced eddy current heat is obtained by adding the z-axis spatial angle to the two-dimensional electromagnetic-thermal, thus obtaining some new results by coupling the numerical simulation and computations of the electric field and magnetic induction intensity of the three-dimensional model. Moreover, we have considered the objective function into a weak formulation. The three-dimensional model is then meshed by the finite element method. The electromagnetic-thermal coupling has been numerically computed, and the parametric dependence to the eddy current heating process has been fully studied. Through the numerical simulation with different current densities, frequencies, and distances, the most suitable heat treatment process of U75V rail is obtained.

## 1. Introduction

It has been roughly estimated that the more than 40% of railway problems are due to the increasing number of increased passengers and travel distances. Therefore, in order to ensure the higher speed, load capacity, and smoothness of high-speed rail transportation, together with the safety and stability of the railway, some more strict requirements must be fulfilled. The material [1,2], welding [3,4], heat treatment technology, [5] and forming [6] of railway rails are put forward under more strict requirements.

In the process of rail manufacturing, the thermal stress caused by internal heating increases the loss of rail. Heat treatment technology ensures rail quality by eliminating thermal stress [7,8,9]. Eddy current heating technology, which has the advantages of high performance and environmental protection, has been widely applied [10,11]. In this paper, the eddy current heating technology is applied to the process of rail heat treatment. However, during the heating process, the heating temperature affects the heat treatment [12,13]. Therefore, in order to improve the welding quality of the rail, the heating temperature must be controlled strictly. At present, the application of eddy current heating in the study of rail heat treatment adopts a two-dimensional model analysis [14,15].

However, the calculation of temperature in the two-dimensional model is carried out only in the axial direction, by ignoring the computation along the longitudinal directions. A large number of experimental data show that the two-dimensional model cannot completely solve the current problem [16,17]. Therefore, we propose a three-dimensional electromagnetic-thermal coupling model of rail welding. In the following paper, the electromagnetic and temperature field are obtained by combining electromagnetic-thermal coupling with the numerical simulation of rail normalizing. In our model, the thermal properties of various parameters during the process of induction heating [18,19] are fully taken into consideration. The three-dimensional numerical calculation process of eddy current normalizing of U75V rail is designed, and the parameters of the 3d model are compared. This model not only provides theoretical basis for multi-parameter selection under complex process conditions, but also has positive significance for improving the safety and reliability of rail welding quality.

In addition, because of the large investment in hardware testing and the high operating cost, the multiple test verification of the eddy current heating application process [20,21] makes the computer numerical simulation preferable in order to simulate the electric vortex positive heat treatment of the steel rail on the high-speed rail track [22,23].

In this paper, changes in the induced magnetic field, the eddy current, and the rail temperature during the eddy current normalizing heat treatment of the U75V rail are studied. The finite element numerical simulation method [24,25] is used for numerical simulation of the eddy current heat treatment process of the rail. The finite element differential equation in the mesh division is also established by taking into account the Maxwell equation [26,27] in electromagnetic theory and the Fourier heat transfer law [28,29] in the heat transfer theory. The numerical simulation results are analyzed by the magnetic induction and temperature changes of the U75V rail eddy current normalized heat treatment under different conditions.

The structure of this paper is as follows. Section 2 introduces the parameters and the numerical calculation method of the three-dimensional model. Section 3 shows the finite element mesh generation. Section 4 gives the process of rail heat treatment. Section 5 analyzes the results of heat treatment of steel rail under different conditions.

## 2. Three-Dimensional Rail Welding Model

### 2.1. Establish the Three-Dimensional Rail Welding Model

**Figure 1:**
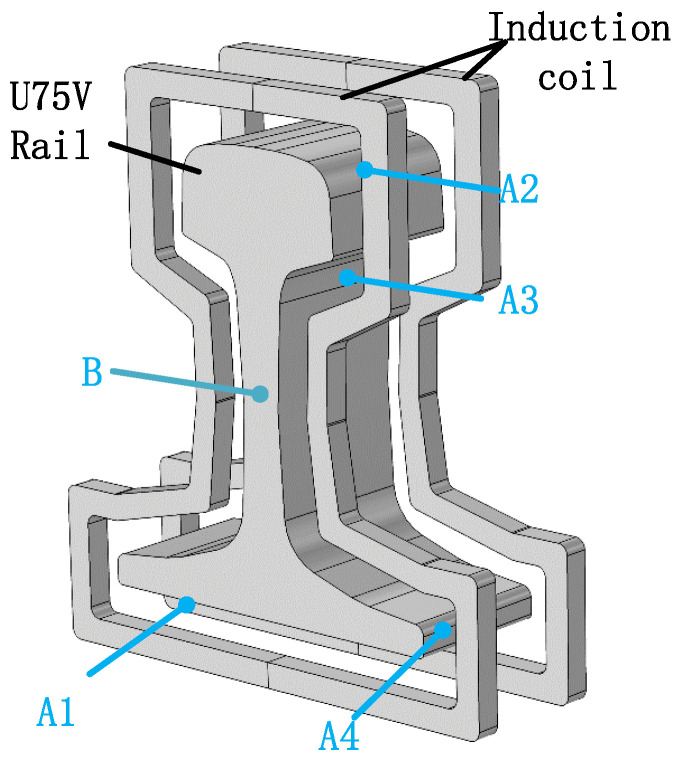
The three-dimensional rail welding model.

The research object of this paper is U75V rail steel. The actual parameters of the rail are the following [13,30]: track length is 50 mm, track density 7894 kg/m^3^, induction coil section inner radius 110 mm, outer radius 120 mm, and induction coil width 50 mm. Five points of A1, A2, A3, A4, B on the rail are picked to measure the temperature of the equipment (Figure 1). The frequency is 1600 Hz, the current density is 2 × 10^7^ A/m^3^, and air domain size 300 mm × 300 mm × 300 mm. The initial temperature is 293.15 K, the heating time is 115 s under the circular induction coil, and 60 s under the contour induction coil. The relationship between material properties and temperature and the influence of latent heat on temperature are considered (Table 1) [31]. The sequential electromagnetic-thermal coupling method is used to simulate the heat treatment process of the rail. Firstly, the electromagnetic field is analyzed, and then the Joule heat produced by induced eddy current is calculated as the heat source of the thermal analysis.

**Figure 2:**
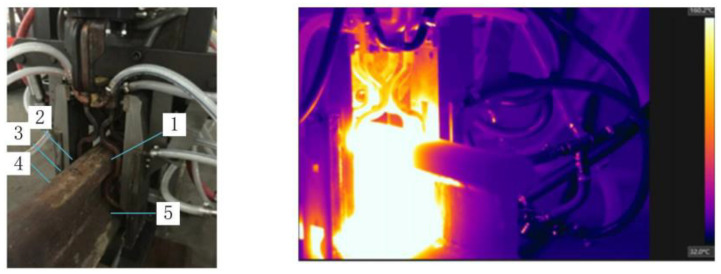
The hardware experiment device.

Through the hardware experiment of Professor Szychta Leszek from Kazimierz Pulaski Technical University [32], the feasibility of the simulation experiment is verified, which used a standard rail induction welding test bench, i.e., USCT6785. The hardware test is shown in Figure 2. The rail model is U75V, the temperature of the experimental environment is 20 °C, the distance between the coil and the rail is 5 cm, the coil current density is 2 × 10^7^ A/m^3^, and the frequency is 1600 Hz. There are five positions from 1 to 5 on the U75V rail for the temperature sensor, corresponding to A1 to B in Figure 1. When the experiment is performed, the five temperature sensors feedback the temperature change in real time. This experiment was completed in July 2014, and the final temperature results of the experiment are published on the website [33].

### 2.2. Three-Dimensional Rail Welding Model Numerical Calculation

In this paper, the three-dimensional rail welding model is proposed by combining Maxwell equations with the basic theory of the Fourier heat transfer equation [26,29], and a complete calculation method of a three-dimensional physical vector is designed. In other words, the changes of temperature, electric field, and magnetic induction intensity of some special points in the three-dimensional space position (A1–A4, B) on the rail are calculated. Taking point A1 as the representative, this paper introduces the calculation process of three-dimensional numerical value in detail.

#### 2.2.1. Calculation of Three-Dimensional Electric Field Strength

**Figure 3:**
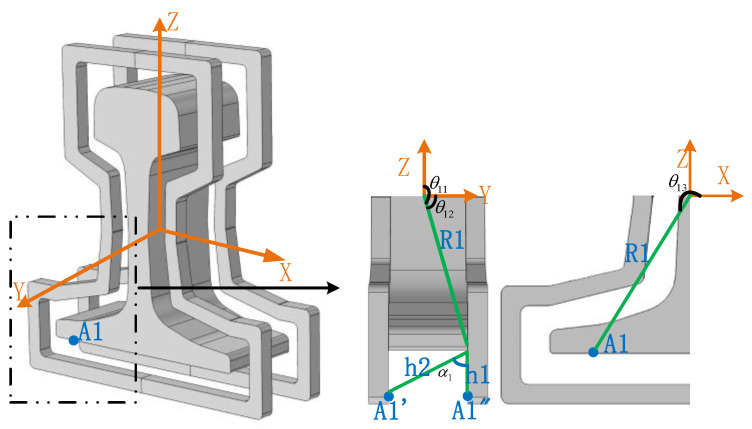
Electric field intensity of A1.

The position of A1 in the model space is shown in Figure 3. The influence of various directions on the electric field intensity of A1 should be considered, which is generated by the combined action of two coils in a circular double-turn single coil. The electric field line passes through the air gap between the coil and the rail, generating an electric field at A1.

The amount of charge at A1′ and A1″ is shown below:

(1) $$q=\frac{J}{s*t}$$

where *J* is the current density, s is 1*m*^2^ area, and t is 1*s* time interval.

Due to electrostatic shielding, the electric field intensity inside the coil is 0, and the electric field intensity outside the coil is equal. The electric field intensity at A1 is calculated by means of the Ampere loop law [34] and Faraday’s electromagnetic induction law [29] in Maxwell equations:

(2) $$\begin{array}{ll} E_{A1} & =\int_{\theta_{11}}^{\theta_{12}}\frac{q_{A1^{\prime }}\cos\theta_{13}}{2\pi \varepsilon_{air}h2}d\theta_{13}+\int_{\theta_{11}}^{\theta_{12}}\frac{q_{A1^{\prime \prime }}\cos\theta_{13}}{2\pi \varepsilon_{air}h2}d\theta_{13}-\int_{\theta_{11}}^{\theta_{12}}\frac{q_{A1^{\prime }}q_{A1^{\prime \prime }}}{2\pi \varepsilon_{air}R1\cos\theta_{13}}*h1\cos\theta_{13}d\theta_{13}\\ & =\frac{q_{A1^{\prime }}}{2\pi \varepsilon_{air}h2}\left(\sin\theta_{12}-\sin\theta_{11}\right)+\frac{q_{A1^{\prime \prime }}}{2\pi \varepsilon_{air}h2}\left(\sin\theta_{12}-\sin\theta_{11}\right)-\frac{q_{A1^{\prime }}q_{A1^{\prime \prime }}}{2\pi \varepsilon_{air}R1}*h1\left(\theta_{12}-\theta_{11}\right) \end{array}$$

where parameters are indicated in Figure 3, and $\varepsilon_{air}$ is the dielectric constant in air, and its value is $6.72\times 10^{-12}F/m$, which has been modified to $\varepsilon_{air}$.

#### 2.2.2. Calculation of Magnetic Induction in Three-Dimensional Model

**Figure 4:**
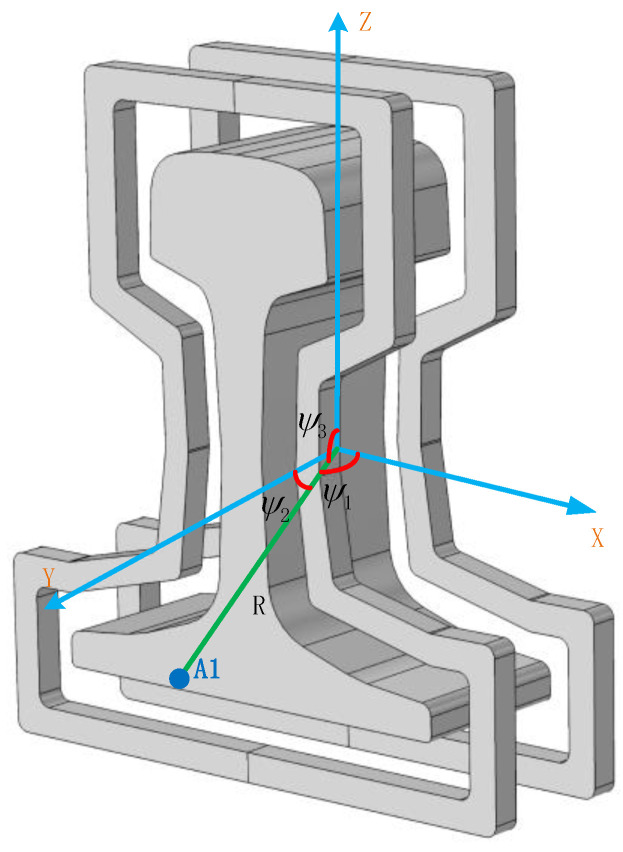
Magnetic induction at A1.

By calculating the electric field strength value obtained from the position of A1, the magnetic induction intensity of the induced magnetic field at A1 can be derived, according to the Gaussian law of electric flux and the Gaussian flux law in Maxwell equations [35]. The magnetic induction field of A1 is shown in Figure 4:

(3) $$\begin{array}{ll} B_{A1}= & \frac{\mu_{0}E_{A1}\sin\psi_{2}}{2R^{2}}\left(\cos\psi_{3}-\cos\psi_{1}\right)\;+\frac{\mu_{0}E_{A1}\sin\psi_{1}}{2R^{2}}\left(\sin\psi_{3}-\sin\psi_{2}\right)\\ & +\frac{\mu_{0}E_{A1}\tan\psi_{3}}{2R^{2}}\left(\tan\psi_{2}-\tan\psi_{1}\right) \end{array}$$

where $\mu_{0}$ is the permeability in a vacuum, its value is $4\times 10^{-7}H/m$, and R is the length of point A1 from the origin of the coordinate system.

## 3. Three-Dimensional Meshing Mathematical Model

In the numerical simulation of the U75V rail, mesh generation of the three-dimensional model is an important part of the whole process. Because of the U75V orbital electric field intensity *E*, the magnetic induction intensity *B* and induced eddy current *J_A_*_1_ need to consider the influence of each point in the entire electromagnetic field. However, in the actual numerical calculation, the points in the model space exceed the computational limit. In this case, the finite element mesh generation method [24,36] is used in the numerical calculation to convert infinite points into finite points, and then each node after grid planning is calculated.

### 3.1. Basic Cell Grid

**Figure 5:**
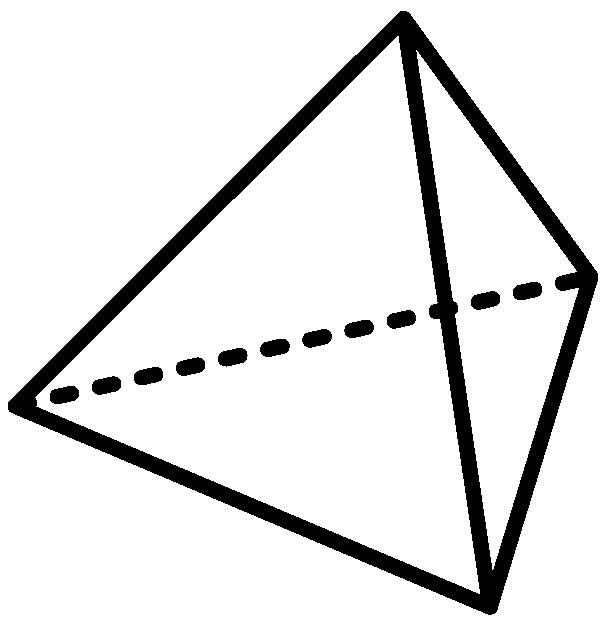
Regular tetrahedral cell.

The basic cell in the three-dimensional finite element meshing method is a regular tetrahedron shape (see Figure 5), and displacement changes in the element and meshing are linear and nonlinear, respectively. Therefore, the amount of calculation in the division process is greatly simplified.

Constructing the displacement model is the first step to solve the problem by finite element analysis. The displacement mode function ϕ=[u,v] expresses the displacement of any point in the element with the displacement value at the node of the element:

(4) $$\phi =\left[u,v\right]=\left\{ \begin{array}{l} u\left(x,y,z\right)=a_{1}+a_{2}x+a_{3}y+a_{4}z\\ v\left(x,y,z\right)=a_{5}+a_{6}x+a_{7}y+a_{8}z \end{array}\right.$$

**Figure 6:**
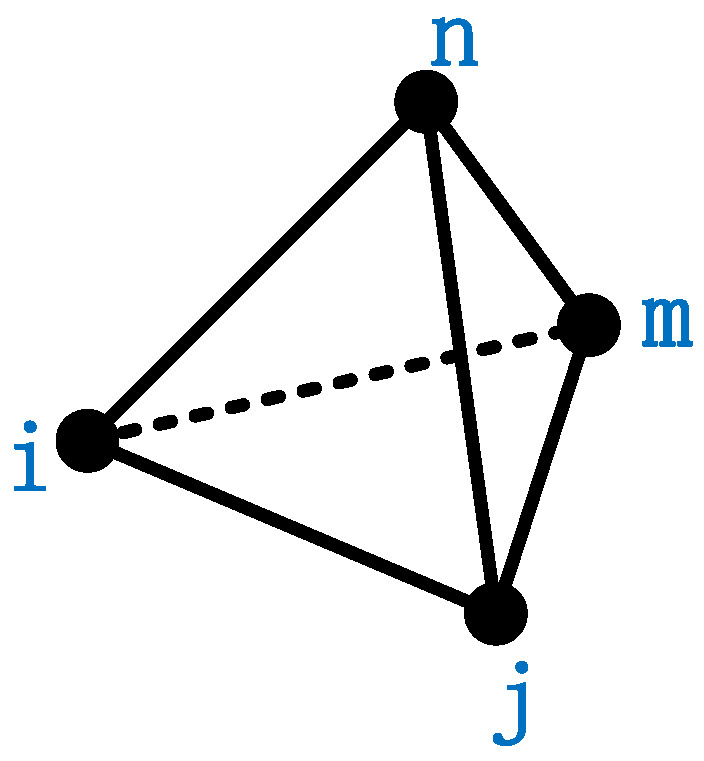
Regular tetrahedral cell vertex order.

The displacement of any point on a regular tetrahedral cell is the position function of that point on the x-axis, y-axis, and z-axis. The four points on the regular tetrahedral cell are numbered counterclockwise (as shown in Figure 6), that is, the coordinates and displacement values of the *i* node, *j* node, *m* node, and *n* node are $\left(x_{i},y_{i},z_{i}\right)$ and $u_{i},v_{i}$, $(x_{i},y_{i},z_{i})$ and $u_{j},v_{j}$, $(x_{m},y_{m},z_{m})$ and $u_{m},v_{m}$, $(x_{n},y_{n},z_{n})$ and $u_{n},v_{n}$.

Substitute the coordinates and displacement value of the node into the displacement mode to get a system of equations about: $a_{i}$:

(5) $$\left[\begin{array}{l} a_{1}\\ a_{2}\\ a_{3}\\ a_{4} \end{array}\right]={\left[\begin{array}{cccc} 1 & x_{i} & y_{i} & z_{i}\\ 1 & x_{j} & y_{j} & z_{j}\\ 1 & x_{m} & y_{m} & z_{m}\\ 1 & x_{n} & y_{n} & z_{n} \end{array}\right]}^{-1}\left[\begin{array}{l} u_{i}\\ u_{j}\\ u_{m}\\ u_{n} \end{array}\right]\;\;=\left[\begin{array}{cccc} \alpha_{i} & \alpha_{j} & \alpha_{m} & \alpha_{n}\\ \beta_{i} & \beta_{j} & \beta_{m} & \beta_{n}\\ \gamma_{i} & \gamma_{j} & \gamma_{m} & \gamma_{n}\\ \tau_{i} & \tau_{j} & \tau_{m} & \tau_{n} \end{array}\right]\left[\begin{array}{l} u_{i}\\ u_{j}\\ u_{m}\\ u_{n} \end{array}\right]$$

(6) $$\left\{ \begin{array}{l} a_{1}=\alpha_{i}u_{i}+\alpha_{j}u_{j}+\alpha_{m}u_{m}+\alpha_{n}u_{n}\\ a_{2}=\beta_{i}u_{i}+\beta_{j}u_{j}+\beta_{m}u_{m}+\beta_{n}u_{n}\\ a_{3}=\gamma_{i}u_{i}+\gamma_{j}u_{j}+\gamma_{m}u_{m}+\gamma_{n}u_{n}\\ a_{4}=\tau_{i}u_{i}+\tau_{j}u_{j}+\tau_{m}u_{m}+\tau_{n}u_{n} \end{array}\right.$$

where α, β, γ, τ, are the node coordinates of the regular tetrahedral cell. The same can be said about $a_{5}$, $a_{6}$, $a_{7}$, $a_{8}$. The value of $a_{i}$ is substituted into the displacement mode function to obtain:

(7) $$\phi =\left[u,v\right]=\left\{ \begin{array}{ll} u\left(x,y,z\right)= & \left(\alpha_{i}+\beta_{i}x+\gamma_{i}y+\tau_{i}z\right)u_{i}+\left(\alpha_{j}+\beta_{j}x+\gamma_{j}y+\tau_{j}z\right)u_{j}\\ & +\left(\alpha_{m}+\beta_{m}x+\gamma_{m}y+\tau_{m}z\right)u_{m}+\left(\alpha_{n}+\beta_{n}x+\gamma_{n}y+\tau_{n}z\right)u_{n}\\ v\left(x,y,z\right)= & \left(\alpha_{i}+\beta_{i}x+\gamma_{i}y+\tau_{i}z\right)v_{i}+\left(\alpha_{j}+\beta_{j}x+\gamma_{j}y+\tau_{j}z\right)v_{j}\\ & \;+\left(\alpha_{m}+\beta_{m}x+\gamma_{m}y+\tau_{m}z\right)v_{m}+\left(\alpha_{n}+\beta_{n}x+\gamma_{n}y+\tau_{n}z\right)v_{n} \end{array}\right.$$

### 3.2. Three-Dimensional Eddy Current Finite Element Model

In three-dimensional eddy current heating, the physical field is usually divided into vortex $\Omega_{1}$ and non-vortex $\Omega_{2}$ regions, and the boundary Γ is divided into an outer boundary $\Gamma_{B},\Gamma_{H}$ and an inner boundary $\Gamma_{0}$. In the non-vortex $\Omega_{2}$, the power supply current density and eddy current density are $J_{s}\neq 0$ and $J_{e}=0$ respectively, so only the magnetic field needs to be considered. In vortex $\Omega_{1}$, the electric and magnetic fields are considered simultaneously. According to the Maxwell equations, the field equations and boundary conditions of the eddy current field can be expressed as follows.

The field equation of $\Omega_{1}$:

(8) $$\left\{ \begin{array}{l} \nabla \times \left(v\nabla \times A\right)-\nabla \left(v\nabla \cdot A\right)+\sigma \frac{\partial A}{\partial t}+\sigma \nabla \varphi =0\\ \nabla \cdot \left(-\sigma \frac{\partial A}{\partial t}-\sigma \nabla \varphi \right)=0 \end{array}\right.$$

The field equation of $\Omega_{2}$:

(9) $$\nabla \times \left(v\nabla \times A\right)-\nabla \left(v\nabla \cdot A\right)=J_{s}$$

The boundary condition of $\Gamma_{B}$:

(10) n·∇×A=0

The boundary condition of $\Gamma_{H}$:

(11) (v∇×A)×n=0

The boundary condition of $\Gamma_{1}$:

(12) $$\left\{ \begin{array}{l} A_{1}=A_{2}\\ v_{1}\nabla \cdot A_{1}=v_{2}\nabla \cdot A_{2}\\ v_{1}\nabla \times A_{1}\times n_{1}=v_{2}\nabla \cdot A_{2}\times n_{1}\\ \nabla \cdot \left(-\sigma \frac{\partial A}{\partial t}-\sigma \nabla \varphi \right)=0 \end{array}\right.$$

where A is vector magnetic potential, φ is scalar potential, σ is conductivity, v is magnetic resistance, ∇× is curl operator, ∇· is divergence operator, and n is the unit normal vector of Γ.

### 3.3. Three-Dimensional Finite Element Meshing

The concept of a weak function is introduced in finite element meshing [10,37]. When the function cannot be solved directly, the integral and curl of the original function are solved, and the form of the weak function equation is added to facilitate the solution of the original function. By transforming Equation (3) into a weak function, the weak function can be obtained as follows:

(13) $$\int_{V}E\omega_{i}dV=\iint_{s}\omega_{i}L_{E}\left(\left[h\right]\varepsilon_{0}\right)ds$$

where *V* is the volume of each cell after division, s is the area of each cell divided, $L_{E}=\int_{V}\frac{q}{4\pi \varepsilon_{0}\left[R\right]}\arctan\left(\theta_{13}-\theta_{12}+\theta_{11}\right)\Lambda dV$ is the weak function equation, $\omega_{i}$ is the auxiliary function, [R] and [h] are the distances from all finite points on the space after grid division to the coordinate origin and the solved point, respectively. $\Lambda =\sum_{i=1}N_{i}\alpha_{i}$ is the test function, $\alpha_{i}$ is the undetermined coefficient, *N* is the number of cells divided, and its value is between 500 and 1000.

**Figure 7:**
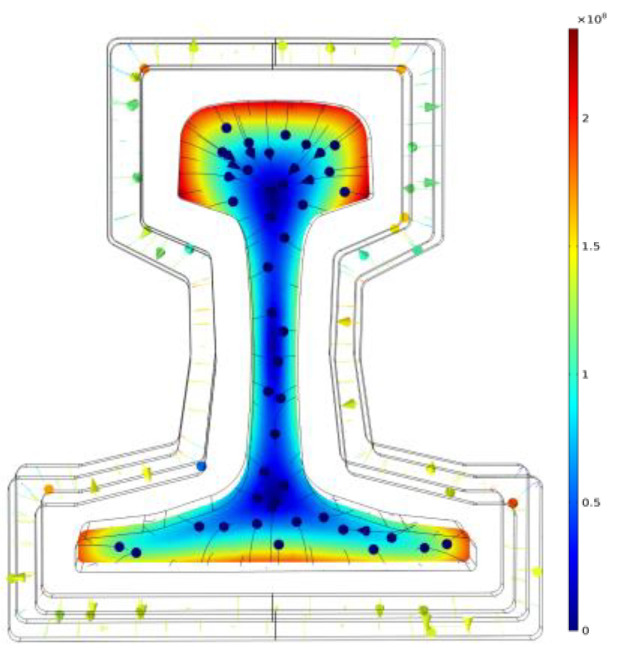
Induced magnetic field distribution.

The electric field generated around the induction coil generates an alternating magnetic field by electromagnetic induction. The magnetic induction intensity, Equation (4), is converted into a weak function as follows:

(14) $$\int_{V}B\omega_{i}dv=\iint_{s}\omega_{i}L_{B}\left(\left[h\right]\right)ds$$

where $L_{B}=\int_{V}\frac{\mu_{0}E}{2{\left[R\right]}^{2}}\mathrm{acttan}\left(\theta_{13}-\theta_{12}+\theta_{11}\right)\wedge dV$ is the weak function equation. During induction heating, an alternating current is fed into the induction coil to produce an induced magnetic field. The magnitude of the induced eddy current in the U75V rail will be subject to the magnetic induction intensity, *B*, generated in the induced magnetic field. Therefore, the magnetic induction line distribution of the induced magnetic field after the finite element mesh division is shown in Figure 7.

The induced current *J_A_*_1_ is a Hamiltonian transformation of the magnetic dipole. Equation (6) is transformed into a weak function in the form of:

(15) $$\int_{V}J_{A1}\omega_{i}dV=\iint_{s}\omega_{i}L_{J_{A}}\left(\left[h\right]\varepsilon_{0}\right)ds$$

**Figure 8:**
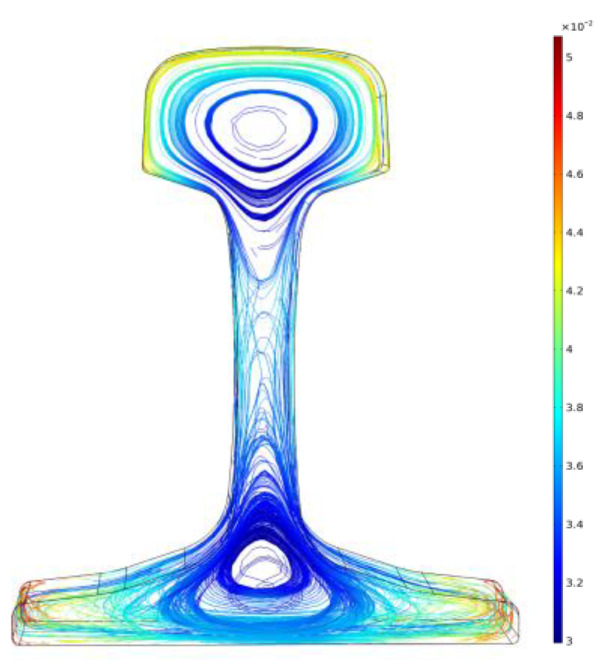
Eddy current distribution.

The induced eddy current generated by the induced magnetic field plays an important role in the rise of the rail temperature. The distribution of the induced eddy current field after finite element mesh division is shown in Figure 8.

When the rail temperature reaches the Curie point, the magnetic characteristics of the rail will disappear and induction heating will no longer be carried out. Therefore, the heating process has two stages: the first stage is induction heating caused by electromagnetic induction, and the second stage is conduction heating, that is, the temperature from high to low [13,38].

During the whole induction heating process, the induction magnetic field is generated when the AC current passes through the induction coil. The magnetic field then creates inductive eddies inside the rail, which generates a lot of heat [39]. Since A1 is on the surface of the U75V orbital, the temperature of A1 is first generated by induction heating. When the temperature reaches the Curie point, the permeability of the current heating layer drops to 1, and the heating mode changes to conduction heating [14,19]. Therefore, the temperature of A1 is obtained by Maxwell equations and Fourier’s law of heat conduction [40,41].

During the induction heating process, the temperature at point A1:

(16) $$q_{A1}=J_{A1}^{2}/\sigma$$

(17) $$T_{1}=Cq_{A1}$$

where σ is the conductivity of the rail, *C* is the specific heat capacity of rail, and $J_{A1}$ is induced current density.

During the conduction heating process, the temperature at A1:

(18) $$T_{2}=\frac{tq_{A1}\sin\theta_{11}}{2k\rho c}e_{x}+\frac{tq_{A1}\cos\theta_{12}}{2k\rho c}e_{y}+\frac{tq_{A1}\tan\theta_{11}}{2k\rho c}e_{z}$$

The temperature that tends to stabilize is *T_end_ = T*_1_ *+ T*_2_.

**Figure 9:**
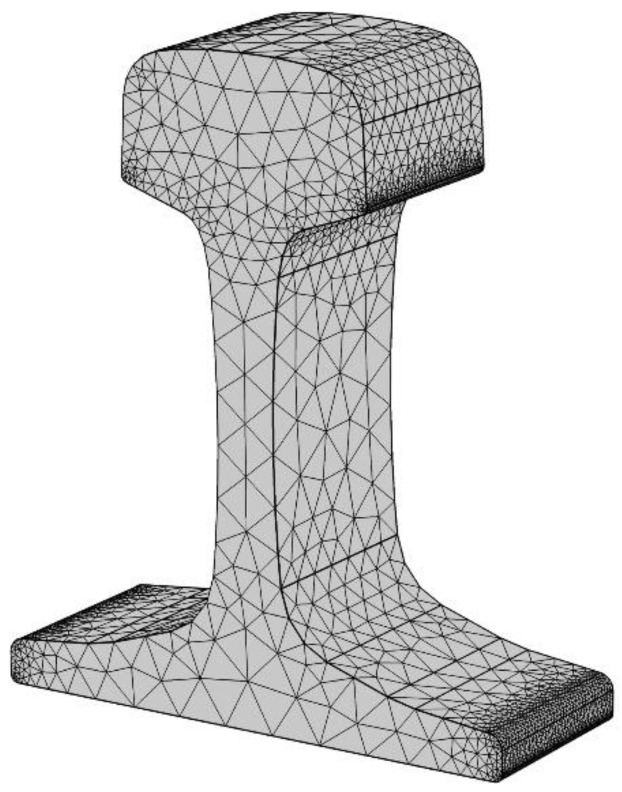
Rail grid planning.

In the finite element division, the Equations (13)–(15) and (18) are iterated continuously by the stable double conjugate gradient method to obtain the optimal solution through the divided grid. Finally, the final meshing value is obtained after 6143 iterations. The finite element analysis grid of induction heat treatment under the circular coil is shown in Figure 9.

## 4. Three-Dimensional Model Simulation Process of Rail Welding

**Figure 10:**
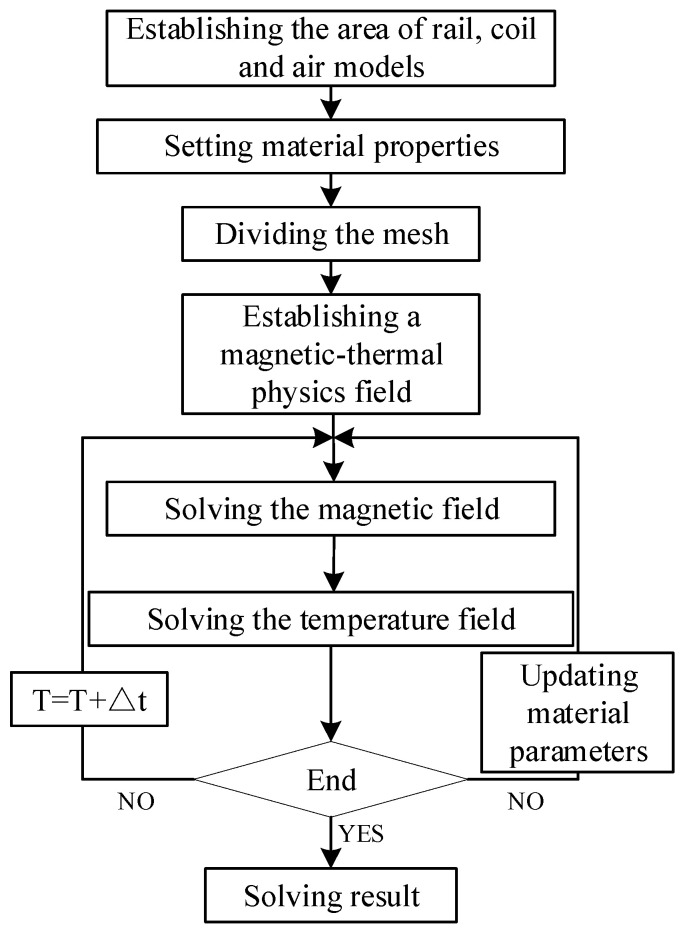
Magnetic-thermal coupling calculation process.

The three-dimensional rail welding model is used, which is solved by the induction heating module. The electromagnetic field is analyzed in the frequency domain and the temperature field by transient analysis. The specific calculation process is shown in Figure 10. To make the calculation result have good convergence, the rails are divided on the mesh division densely, the coils are divided moderately, and the air domain is divided sparsely.

## 5. Results and Discussion

The three-dimensional orbital welding model is established through numerical simulation. By changing the current density and frequency of the induction coil and the distance from the orbit to the induction coil, the influence of each parameter on the temperature can be obtained.

### 5.1. Effect of Current Density on Temperature Field

**Figure 11:**
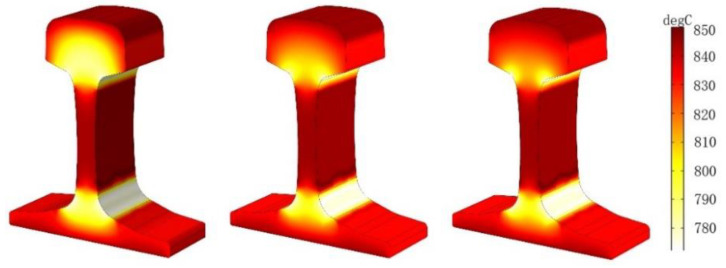
Temperature field of different current density when the rails are constant.

**Figure 12:**
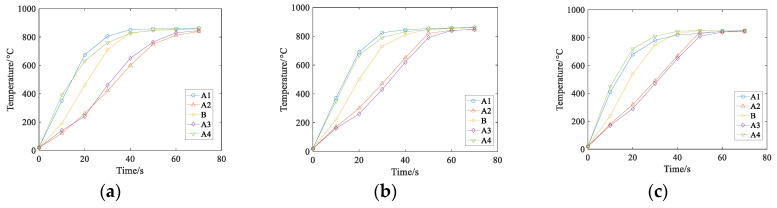
Temperatures of A1–A4 and B at three different current densities. (**a**) $J=2.5\times 10^{5}\;A/m^{3}$, (**b**) $J=3\times 10^{5}\;A/m^{3}$, (**c**) $J=3.5\times 10^{5}\;A/m^{3}$.

The parameters of the rail eddy current heat treatment are set as the distance between the induction coil and the rail, which is 3 cm, and the current frequency is 1500 Hz. However, the density of alternating current flowing into the induction coil will change: *J* = 2.5 × 10^5^ A/m^2^, *J* = 3 × 10^5^ A/m^2^, *J* = 3.5 × 10^5^ A/m^2^, respectively. The three-dimensional cloud chart of the heating temperature field under the coil is shown in Figure 11. The temperature changes at points A1–A4 and B under three different current densities are shown in Figure 12:

As can be seen from Figure 11 and Figure 12, the temperature increase during the heating process increases as the current density of the coil increases if other conditions remain unchanged. The influence of different current densities on the temperature field show that the heating layer at the bottom of the rail gradually reaches the Curie point with the increase of temperature, the temperature at the railhead head also rises to the Curie point, and only the orbital waist temperature continued to rise to 800 ℃. This is because both the railhead and the bottom have warmed up to the Curie point, which further changes the magnetic field around the rail. According to the results of numerical simulation, the heating process of each region is different when the rail is heated by induction. Considering the heating time and stable variation of the coupling field, the current density *J* is selected as *J* = 3.5 × 10^5^ A/m^2^ for the following numerical simulation study.

### 5.2. Effect of Current Frequency on Temperature Field

**Figure 13:**
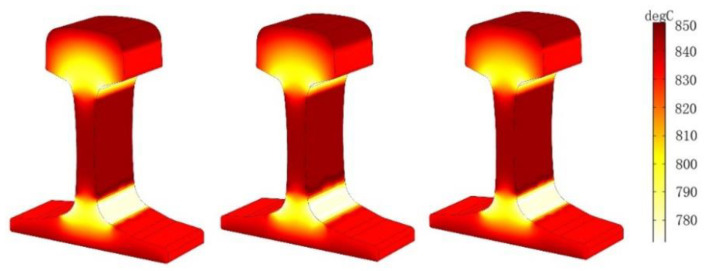
Temperature versus time curve at different current frequencies.

**Figure 14:**
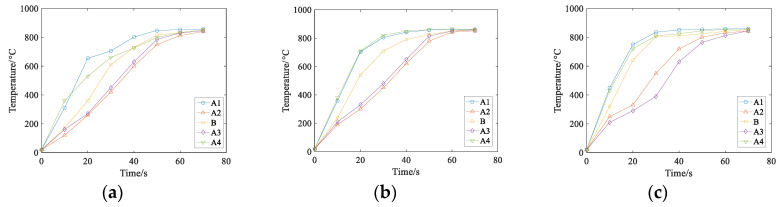
Temperatures of A1–A4 and B at three different current frequencies. (**a**) f=1000 Hz, (**b**)  f=1500 Hz, (**c**)  f=2000 Hz.

In the case, the air gap between the induction coil and the heavy rail end is 3 cm, the current density is *J* = 3.5 × 10^5^ A/m^2^, and the current frequencies of three groups are set as *J* = 1000 Hz, *J* = 2000 Hz, *J* = 3000 Hz. The three-dimensional cloud chart of heating temperature field under the coil is shown in Figure 13, and the temperature changes of points A1–A4 and B under three different current frequencies are shown in Figure 14:

It can be concluded from Figure 13 and Figure 14 that the initial heating layer thins as the current frequency of the input coil increases if the other conditions remain unchanged, and the time required to reach the required temperature is shorter.

Through the numerical simulation calculation in this section, the heating time and the stable variation of the coupling field are considered comprehensively, and the current density *J* is selected as *J* = 3.5 × 10^5^ A/m^2^ for the following numerical simulation study.

### 5.3. Effect of Distance of Coil and Rail on Temperature Field

**Figure 15:**
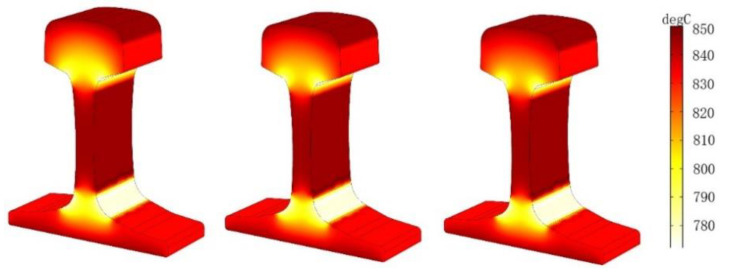
Temperature versus time curve of different distances of induction coils and rail.

**Figure 16:**
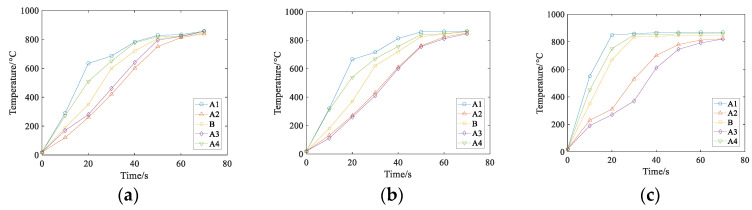
Temperatures of the A1–A4 and B at three different distances. (**a**) 2.5 cm, (**b**) 3 cm, (**c**) 3.5 cm.

Under the condition that the input current density is *J* = 3.5 × 10^5^ A/m^2^, the temperature field is affected not only by the magnitude and frequency of the current passing through the induction coil but also by the distance between the induction coil and the rail. Therefore, the distance between the induction coil and the rail is changed for numerical simulation, which is 2.5 cm, 3 cm, 3.5 cm, respectively. The three-dimensional cloud chart of the heating temperature field under the coil is shown in Figure 15 and the temperature changes of points A1–A4 and B at three different distances are shown in Figure 16:

As can be seen from Figure 15 and Figure 16, if the distance between rail and coil is reduced continuously, the effect of the overall rail-induced positive heat treatment may be affected. When the distance between the coil and the rail is not changed except for other conditions, the temperature of the whole rail will rise very fast as the distance decreases gradually. However, the normalizing process cannot be induced if the distance is too small. Therefore, the rail induction normalizing condition is considered comprehensively: the current size of the induction coil connected is *J* = 3.5 × 10^5^ A/m^2^, the current frequency is 2000 Hz, and the distance between the induction coil and rail is 3 cm as the process parameters of the temperature field simulation analysis.

## 6. Conclusions

In this study, simulation of the heat treatment process for three-dimensional welded rail is achieved, and electromagnetic and temperature fields arising from steel rail are analyzed in the three-dimensional case. The numerical simulation is used to establish a three-dimensional electromagnetic-thermal coupling model of rail eddy current normalizing heat treatment, and a finite element equation is established through the theory of electromagnetics and heat transfer. The original two-dimensional numerical calculation is added to the z-axis rotation angle, and the concept of the weak functions was introduced by the simulation of the established three-dimensional rail welding model on different current densities and frequencies of the induction coil and the distance from the rail to the induction coil. This study provides a quantitative reference and analysis method for studying the formation mechanism of the temperature field in the process of rail induction heating.

## Figures and Tables

**Table 1:** Intrinsic parameters of U75V rail.

Temperature (°C)	Relative Permeability	Resistivity (Ω)	Specific Heat Capacity (J·Kg^−1^°C^−1^)	Coefficient of Thermal Conductivity (W·m^−1^°C^−1^)	Enthalpy (J/m^3^)
25	200	1.84 × 10^−7^	472	93.23	9.16 × 10^7^
100	194.5	2.54 × 10^−7^	480	87.68	3.56 × 10^8^
200	187.6	3.39 × 10^−7^	498	83.35	7.53 × 10^8^
300	181	4.35 × 10^−7^	524	0.44	1.16 × 10^9^
400	169.8	5.41 × 10^−7^	560	78.13	2.12 × 10^9^
500	157.3	6.56 × 10^−7^	615	76.02	2.65 × 10^9^
600	140.8	7.9 × 10^−7^	700	74.16	3.19 × 10^9^
700	100.36	9.49 × 10^−7^	1000	71.98	3.72 × 10^9^
800	1	1.08 × 10^−8^	806	69.66	4.22 × 10^9^
900	1	1.16 × 10^−8^	637	66.49	4.52 × 10^9^
1000	1	1.2 × 10^−8^	602	65.92	5.14 × 10^9^
